# Inhibition of Oxidative Stress by Low-Molecular-Weight Polysaccharides with Various Functional Groups in Skin Fibroblasts

**DOI:** 10.3390/ijms141019399

**Published:** 2013-09-25

**Authors:** Szu-Kai Chen, Chu-Hsi Hsu, Min-Lang Tsai, Rong-Huei Chen, Gregor P. C. Drummen

**Affiliations:** 1 Department of Food Science, National Taiwan Ocean University, 2 Pei-Ning Road, Keelung 20224, Taiwan; E-Mails: skyskchen@gmail.com (S.-K.C.); chuhsi@mail.ypu.edu.tw (C.-H.H.); 2 Department of Food and Beverage Management, Yuanpei University, 306, Yuanpei Street, Hsinchu 30015, Taiwan; 3 Cellular Stress and Ageing Program, Bionanoscience and Bio-Imaging Program, Bio & Nano-Solutions, Helmutstr. 3A, Düsseldorf 40472, Germany; E-Mail: gpcdrummen@bionano-solutions.de

**Keywords:** antioxidant, skin fibroblast, oxidative damage, oxidative stress, ROS, lipid peroxidation, DNA damage, agar, chitosan, starch

## Abstract

The aim of this study was to evaluate the *in cellulo* inhibition of hydrogen-peroxide-induced oxidative stress in skin fibroblasts using different low-molecular-weight polysaccharides (LMPS) prepared from agar (LMAG), chitosan (LMCH) and starch (LMST), which contain various different functional groups (*i.e*., sulfate, amine, and hydroxyl groups). The following parameters were evaluated: cell viability, intracellular oxidant production, lipid peroxidation, and DNA damage. Trolox was used as a positive control in order to allow comparison of the antioxidant efficacies of the various LMPS. The experimentally determined attenuation of oxidative stress by LMPS in skin fibroblasts was: LMCH > LMAG > LMST. The different protection levels of these LMPS may be due to the physic-chemical properties of the LMPS’ functional groups, including electron transfer ability, metal ion chelating capacities, radical stabilizing capacity, and the hydrophobicity of the constituent sugars. The results suggest that LMCH might constitute a novel and potential dermal therapeutic and sun-protective agent.

## 1. Introduction

Free radicals and other oxidizing species induce intra- and extracellular oxidative stress, causing functional decline in cells and tissues [1]. Furthermore, the “free radical theory of aging” purports that damage accumulation as a consequence of oxidative stress is a major cause of aging. Furthermore, oxidative stress is believed to be a primary factor in various degenerative diseases, such as atherosclerosis, inflammation, carcinogenesis, Alzheimer’s disease, and skin aging [2–4]. Oxidizing species can be divided into reactive oxygen species (ROS), such as the superoxide anion (O_2_^•^), the hydroxyl radical (•OH) and hydrogen peroxide (H_2_O_2_), and reactive nitrogen species (RNS), such as nitric oxide (NO•) and peroxynitrite (ONOO^−^) [5]. Solar ultraviolet radiation (UVR) is a potent initiator of ROS generation in the skin [6]. UVR generates superoxide through the activation of NADPH oxidase and respiratory chain reactions [7]. Superoxide is normally neutralized by superoxide dismutase (SOD), which catalyzes the dismutation of superoxide to O_2_ and H_2_O_2_[8]; H_2_O_2_, which is a stable and plasma-membrane permeable oxidant, in turn might form •OH in the presence of transition metal ions through the Fenton reaction [6]. These free radicals initiate the peroxidation of membrane lipids, which leads to radical-chain reactions that culminate in the accumulation of lipid peroxides and a myriad of other degradation products. These in turn are capable of damaging a wide variety of biomolecules [9]. Cells counteract oxidative damage by neutralizing ROS through an extensive antioxidant defense system and/or by repair mechanisms that repair and replace damaged biomolecules [10], thereby protecting organisms against oxidative damage and loss of cellular homeostasis. Generally, the primary enzymatic antioxidant defense system, including catalase (CAT), glutathione peroxidase (GPx), and SOD, is the first-line of defense in ROS detoxification. The nonenzymatic antioxidant defense system consists of low molecular weight antioxidants, including vitamins C and E, coenzyme Q10, and other endogenous small molecules or nutritional compounds. These, not only participate directly in radical scavenging, but also serve as essential cofactors for various enzymes that decrease oxidative stress [11].

Low molecular weight antioxidants can be divided into three categories, depending on the antioxidant mechanism involved [12]: (1) Free-radical terminators, such as butylated hydroxytoluene (BHT), tertiary butylhydroquinone (TBHQ), propyl gallate (PG) and tocopherol (vitamin E), interrupt free-radical chain reactions by donating hydrogen atoms from hydroxyl groups, thereby forming more stable species that do not induce oxidation; (2) Reducing agents or oxygen scavengers, such as ascorbylpalmitate, sulfite, ascorbic acid, erythorbic acid, and glucose oxidase, exert their antioxidant mechanism by transferring hydrogen atoms and removing oxygen; (3) Chelating agents, such as citric acid, phosphate and ethylenediaminetetra-acetic acid (EDTA), form stable coordination complexes with pro-oxidative metal ions, such as iron (Fe^2+^) and copper (Cu^2+^) ions and delay the oxidation process.

In recent years, polysaccharides have been demonstrated to scavenge free radicals *in vitro* and are increasingly used as antioxidants for the prevention of oxidative damage in foods [13] and living organisms [14]. The antioxidant activity of polysaccharides depends on several structural parameters, such as molecular weight [15,16], type and position of the functional groups [17] (e.g., hydroxyl [18], sulfate [17,19–21], amino [17,18], carboxyl [21], and phosphate groups [20,22]), the type of saccharide and glycosidic branching [23], and the degree of substitution [24].

Chitosan, a cationic polysaccharide in acidic solution, is made up of d-glucosamine and *N*-acetylglucosamine linked through β-1,4glycosidic linkages. It is produced by the deacetylation of chitin obtained from crab and shrimp shells. Chitosan has several reactive groups such as –OH and –NH_2_, which can react with many different compounds [25]. The antioxidant activity of chitosan depends on the degree of deacetylation [15] and its molecular weight [13,15,25]. Agar, extracted from red seaweed, is composed of agarose and agaropectin [26]. Agarose, a neutral polysaccharide, consists of β-d-galactose and 3,6-anhydro-α-l-galactose. Agaropectin, a negative-charged sulfated polysaccharide, consists of β-1,3-glycosidically linked d-galactose units, some of which are sulfated at the C-4 and C-6 position [27]. The antioxidant activities of agar are related to molecular weight and to the sulfate group content [16].

In a previous study [17], we used chemical antioxidant capacity assays to test the ROS scavenging capacities of LMPS *in vitro*. However, the antioxidant mechanisms in organisms, tissues, and cells are more complex than those occurring in a clean chemical environment. Consequently the antioxidative capacity of these compounds might differ significantly *in vivo*, exactly because there are many endogenous and exogenous factors, such as ultraviolet light, metal ions, chemical reactions, intracellular antioxidant enzymes and cytokines, which influence the extent of ROS generation and oxidative stress induction in cells and tissues. The purpose of this study was, therefore, to use a cell culture model to understand the antioxidant capacities of LMPS in a more complex biological setting. We evaluated the inhibitory effect of low-molecular-weight polysaccharides prepared from agar, chitosan, and starch in skin fibroblasts, and characterized these according to their sulfate, amine and hydroxyl functional groups. Their efficacy to counteract oxidative stress was evaluated by measuring cytotoxicity, intracellular ROS production, lipid peroxidation, and DNA damage, induced by hydrogen peroxide. Electron transfer and chelating metal ions limiting ROS production were used to explain the different antioxidant activities and to elucidate the relationship between structure and activity.

## 2. Results

### 2.1. Characterization of LMPS

Prior to use, the molecular and structural properties of the LMPS were characterized. The properties of the saccharides used were as follows: molecular weights (MW) of LMAG, LMCH, and LMST were 3573, 3767, and 3643 Da, respectively. The degrees of polymerization of LMAG, LMCH, and LMST were 20, 22, and 22, respectively. The degree of deacetylation (DD) of LMCH was 89.6% ± 7.1%. The sulfate content of LMAG was 11.8% ± 0.3%.

### 2.2. The Intrinsic Cytotoxicity of LMPS

**Figure 1:**
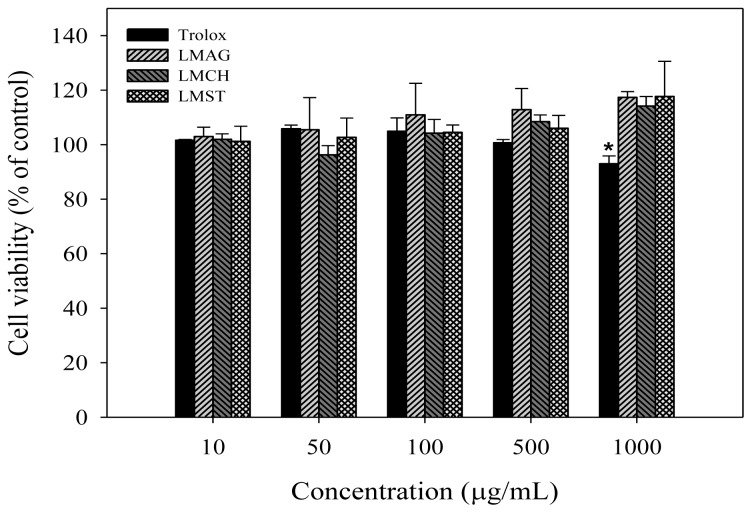
The cytotoxic effects of low-molecular-weight agar (LMAG), low-molecular-weight chitosan (LMCH), low-molecular-weight starch (LMST) and trolox on Hs68 cell viability. Data are expressed as mean values ± S.D. of triplicate experiments. *p < 0.05 (LM-polysaccharides *vs.* trolox).

The effects of LMAG, LMCH, LMST, and trolox on the viability of Hs68 cells *per se* are depicted in Figure 1. Trolox, a water-soluble derivative of vitamin E, was used as a standard antioxidant in this study for comparison reasons. Figure 1 showed that after 24 h of treatment with different concentrations of LMAG, LMCH, LMST, and trolox, the viabilities of Hs68 cells were not significantly different between the samples and the control, and therefore, no intrinsic cytotoxicity was observed. Please note that the cell viability of trolox decreased to 93% at the highest concentration of 1000 μg/mL, but this change was not significant compared with the control.

### 2.3. Effects of LMPS on the Viability of H_2_O_2_-Treated Cells

**Figure 2:**
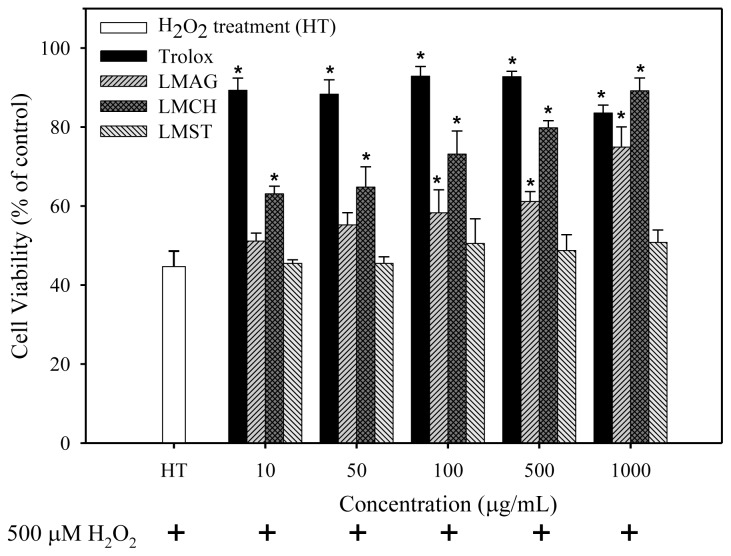
The effects of low-molecular-weight agar (LMAG), low-molecular-weight chitosan (LMCH), low-molecular-weight starch (LMST), and trolox on the viability of Hs68 cells in response to 24 h treatment with 500 μM H_2_O_2_. Data are expressed as mean values ± S.D. of triplicate experiments. *p < 0.05 (treatment *vs.* HT); HT = H_2_O_2_ treatment.

To assess the capacity of the LMPS to attenuate oxidative stress, cell viability was determined when additionally incubating Hs68 cells with H_2_O_2_. As shown in Figure 2, oxidative stress induction with 500 μM H_2_O_2_ (HT) in the absence of LMPS or trolox resulted in a survival rate of approximately 45% after 24 h exposure. Conversely, when incubating LMAG and LMCH, a dose-dependent recovery in cell viability was observed: ~75% and ~89% cell viability at 1000 μg/mL, respectively. The figure also shows that trolox was unable to completely ameliorate the effects of oxidative stress and the maximum viability of cells treated with trolox remained at ~85% from 10 to 1000 μg/mL. Furthermore, the capacity of trolox to protect Hs68 cells against H_2_O_2_ injury was nearly 10% higher at 1000 μg/mL compared with LMAG, but ~4% lower than LMCH at 1000 μg/mL. Interestingly, LMST had virtually no effect on cell viability over the complete concentration range tested, and significant differences compared with HT were in effect absent.

### 2.4. Inhibition of Intracellular ROS Production

**Figure 3:**
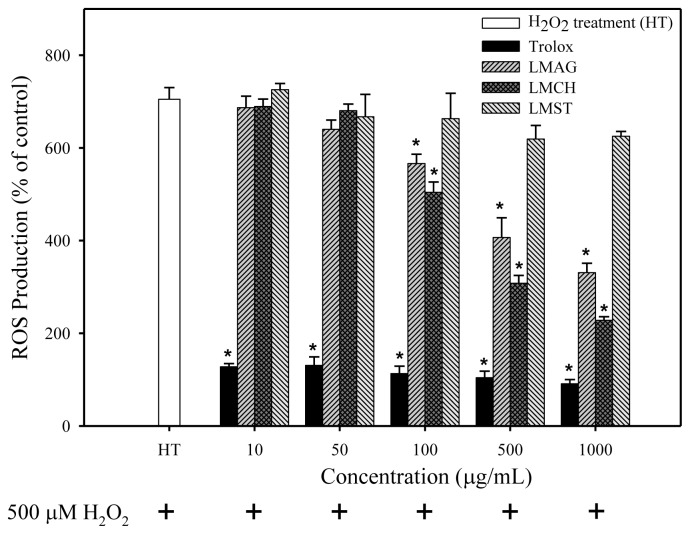
The effects of low-molecular-weight agar (LMAG), low-molecular-weight chitosan (LMCH), low-molecular-weight starch (LMST), and trolox on the production of intracellular reactive oxygen species (ROS) in Hs68 cells after 30 min treatment with 500 μM H_2_O_2_. Data are expressed as mean values ± S.D. of triplicate experiments. *p < 0.05 (treatment *vs*. HT); HT = H_2_O_2_ treatment.

Figure 3 depicts the effects of LMAG, LMCH, LMST, and trolox on intracellular ROS production in H_2_O_2_-treated Hs68 cells. The results of HT showed that the intracellular ROS production was increased 7-fold under treatment with 500 μM H_2_O_2_. Pretreatment with trolox decreased ROS production concentration-dependently over the full concentration range by an initial factor of ~6 to ~7.8 at the highest concentration. Conversely, pretreatment with LMAG and LMCH had no inhibitory effect on ROS production from 10 to 50 μg/mL, but a clear concentration-dependent attenuation of ROS production occurred from 100 to 1000 μg/mL. LMST showed trivial inhibitory activity at all concentrations tested, in concordance with the cell viability results. Overall, the results showed a significant and concentration-dependent antioxidant activity of LMAG and LMCH, albeit that the maximum effect at the highest concentration was still a factor ~3.6 lower than trolox. Interestingly, at low concentration, a lag-phase in the effect was discernible.

### 2.5. Inhibition of Cellular Lipid Peroxidation

**Figure 4:**
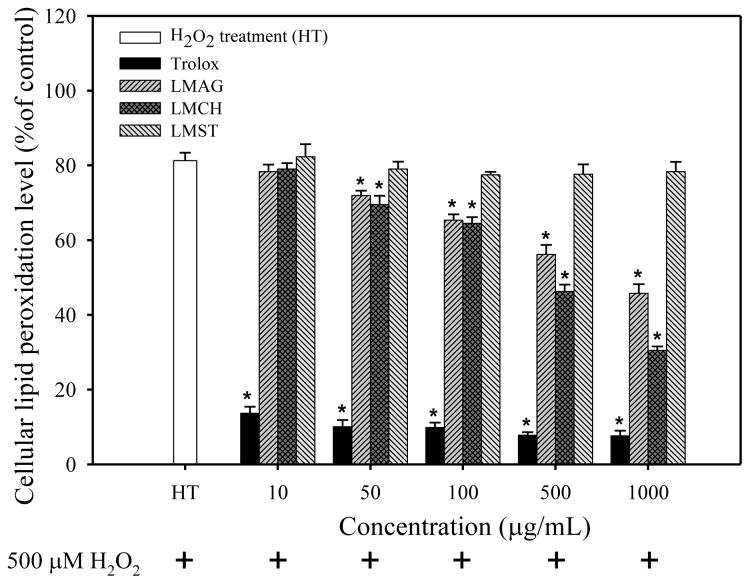
The effects of low-molecular-weight agar (LMAG), low-molecular-weight chitosan (LMCH), low-molecular-weight starch (LMST), and trolox on the cellular lipid peroxidation level of Hs68 cells after 24 h treatment with 500 μM H_2_O_2_. Data are expressed as mean values ± S.D. of triplicate experiments. *p < 0.05 (treatment *vs*. HT); HT = H_2_O_2_ treatment.

Since the polyunsaturated fatty acids in biomembranes lipids are opportune targets for ROS, we evaluated the ability of the LMPS to attenuate lipid peroxidation in Hs68 cells, challenged for 24 h with 500 μM H_2_O_2_. Figure 4 depicts the effects of the LMPS and trolox on cellular lipid peroxidation in HT Hs68 cells. The intrinsic cellular lipid peroxidation level in Hs68 cells was about 81% after exposure to H_2_O_2_ for 24 h. Conversely, and in concordance with the ROS measurements, preincubation with LMAG and LMCH resulted in a concentration-dependent attenuation of cellular lipid peroxidation, and showed a ~50%–60% reduction in lipid peroxidation level at the highest concentration. The effect of trolox was instantaneous over the whole concentration range and reached a reduction to 10%. However, none of the LMPS tested were able to achieve the protective effect displayed by trolox (still ~4–6 times higher), and at low concentration lagged behind in their effect. The occurrence of a lag-phase in lipid peroxidation and antioxidant treatment is well documented for a large number of antioxidants. As previously noticed in the cell viability and ROS measurements, LMST was unable to attenuate lipid peroxidation and did not significantly differ from HT, which shows its impotency to prevent lipid peroxidation.

### 2.6. Inhibition of DNA Damage

**Figure 5:**
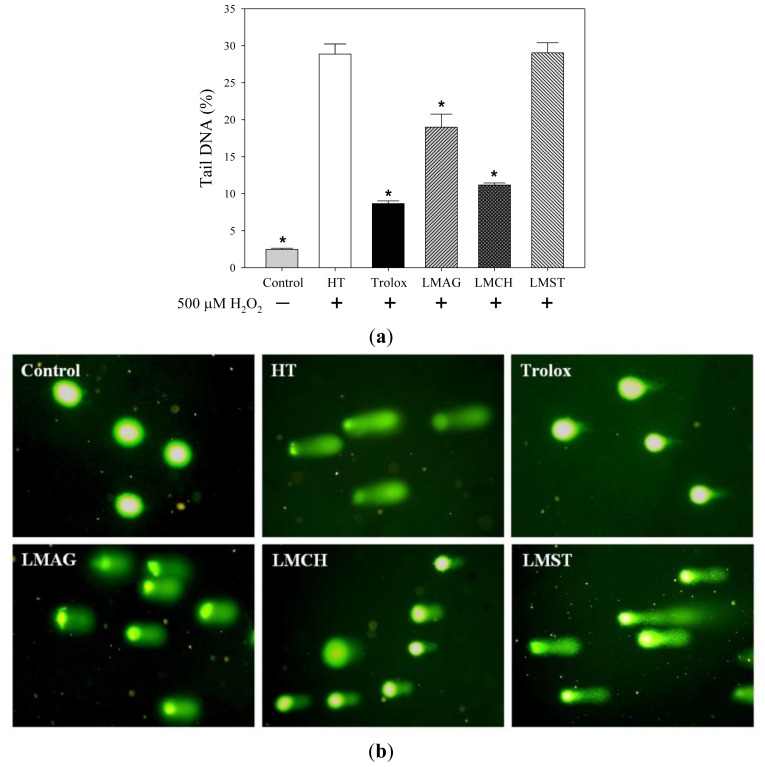
The effects of 1000 μg/mL low-molecular-weight agar (LMAG), low-molecular-weight chitosan (LMCH), low-molecular-weight starch (LMST) and trolox on DNA damage in Hs68 cells after 30 min treatment with 500 μM H_2_O_2_. (**a**) Tail DNA (%) was calculated from the measurement of 50 cells per slide by Comet-Score image analysis. Data are expressed as mean values ± S.D. of three slides. *p < 0.05 (treatment *vs*. HT); HT = H_2_O_2_ treatment; (**b**) Representative Comet images (SYBR^®^ Green I) of individual Hs68 cells, acquired with a fluorescence microscope equipped with a digital camera.

To determine the capacity of the various LMPS to prevent damage to nuclear DNA, Comet evaluations were performed as shown in Figure 5. The result in Figure 5A showed that nearly 30% DNA strand breaks occurred when challenged with 500 μM H_2_O_2_ for 30 min (tail increased from 2.5% in control cells to 28.9% in HT cells). Pretreatment with trolox, LMAG, or LMCH reduced the amount of tail DNA by a factor of ~3, ~1.5, and ~2.6, respectively, at 1000 μg/mL (Figure 5A,B). In contrast, Hs68 cells pretreated with LMCH at the highest concentration showed no significant difference with HT. Furthermore, none of the compounds tested were able to reduce DNA strand breaks to control levels; preincubation with the most powerful antioxidant tested here, trolox, still resulted in a 3.5 fold increase in strand breaks and tail DNA (Figure 5B). Finally, Figure 5B also shows that in the control cells, virtually no tail DNA was detected. In general, these results are in good agreement with the results reported above.

## 3. Discussion

When ROS production overwhelms the cellular endogenous antioxidant capacity, an increase in lipid peroxidation and oxidative DNA injury occurs [28]. Lipid peroxidation, a complex radical chain reaction leading to oxidation of cell membrane lipids, is considered a critical mechanism of injury that occurs in cells during oxidative stress [29]. Reactive lipid peroxidation products, such as hydroperoxides, lipid peroxyl and alkoxyl radicals [28], and aldehydic fatty acid derivatives, e.g., malon dialdehyde and 4-hydroxynonenal, not only cause cellular membrane damage, but also damage and/or modify proteins, DNA, and other biomolecules. Transition metal ions, such as ferrous (Fe^2+^) and cuprous (Cu^+^) ions, are able to induce the formation of reactive lipid species from lipid (hydro)peroxides [12]. They act as catalysts in the propagation steps of the lipid peroxidation chain reaction, which culminates in an amplification of the damage induced by the initiating radical. Antioxidants counteract ROS by either terminating free radicals, directly reducing oxidizing species or by scavenging oxygen, and by chelating transition metal ions, thereby preventing Fenton reactions from occurring [12]; often antioxidants combine two or more properties in the same molecule.

The antioxidant mechanisms of a variety of carbohydrates have been studied extensively. Ji *et al*. [25] reported that low-molecular-weight chitosans, having a compact structure and many free hydroxyl and amino groups, could react directly with free radicals. This might be one of the reasons why the antioxidant activities of low-molecular-weight saccharides were stronger than those of high-molecular-weight saccharides. Chitosan oligosaccharides prevented oxidative damage to cell membrane lipids and nuclear chromatin, induced by hydrogen peroxide, thereby alleviating cell apoptosis [30,31]. This effect was likely due to chitosan oligosaccharides’ ability to limit hydroxyl radicals (•OH) generated by Fenton reactions via metal ion-catalyzed conversion from H_2_O_2_[30,31]. Sun and co-workers [24] showed that the carboxymethyl group enhanced the electron cloud density of active hydroxyl and amino groups in the *N*-carboxymethyl chitosan oligosaccharide (NCMCOS) chain. This property was responsible for the increased electron-donating activity of NCMCOS. Consequently, NCMCOS’ scavenging ability of the superoxide anion increased when the degree of substitution increased from 0.28 to 0.41. Tsiapali *et al*. [20] described that phosphorylated and sulfated glucan exhibited a higher antioxidant capacity than glucan without any functional groups. Yuan *et al*. [22] showed that the scavenging of DPPH radicals (1,1-diphenyl-2-picrylhydrazyl) by κ-carrageenan oligosaccharide phosphorylated and oversulfated derivatives was stronger than that of κ-carrageenan oligosaccharides *per se*. Campo and collaborators [19] reported that glycosaminoglycans, via the negative charge of the sulfate group, chelate transition metals and limited oxidative injury in skin fibroblast cultures. Ngo *et al*. [30,31] showed that chito-oligosaccharides could chelate metal ions and consequently reduced oxidative damage to DNA and cell membrane lipids. Chen *et al*. [17] reported that the *in vitro* antioxidant capacities of LMCH and LMAG were higher than LMST. The different scavenging capacities may be caused by the combined effects of the electron cloud densities of the functional groups and the hydrophobicities of constituent sugars in LMPS. A higher electron cloud density generally increases the electron-donating activity, whereas a lower hydrophobicity facilitates the accessibility of LMPS to free radicals, which subsequently increases the scavenging effect. The antioxidant activity of LMST was negligible, unlike phenolic compounds, because the internal pyranose ring in starch has no conjugated double bond to donate hydrogen from hydroxyl groups, and subsequently stabilize the radical. The aforementioned studies showed that intrinsic properties, such as molecular weight and the presence and degree of functional groups are the backbone of the biopolymer that determines their antioxidant activity.

Our results showed that up to a concentration of 1000 μg/mL, cell viability in the presence of the LMPS did not significantly deviate from control, and thus no intrinsic cytotoxicity was observed. Similarly, trolox did not induce any cytotoxic effects, albeit that at the highest concentration, an initial, but statistically insignificant, decrease (7%) was observed. Nonetheless, this effect might represent the first sign of pro-oxidant behavior, which might increase the oxidative stress levels [32]. Overall, these results are in good agreement with reports in the literature. For instance, Chen and Yan [33] showed that agaro-oligosaccharides with different degrees of polymerization not only exhibited no cytotoxic effects on human liver cells (L-02) at concentrations from 125 μg/mL to 1 mg/mL, but also stimulated the growth of L-02 cells. Ngo *et al*. [31] reported that chitin oligosaccharides exhibited no cytotoxic effects on human myeloid cells (HL-60) and mouse macrophages (Raw 264.7) in the concentration range of 1 to 1000 μg/mL.

After H_2_O_2_ exposure, cell viabilities of the trolox-treated samples were higher than those of the LMPS-treated ones. Furthermore, ROS production, lipid peroxidation, and DNA damage in trolox-treated Hs68 cells were significantly lower than those of the LMPS-treated ones (results shown in Figures 2–5). Nonetheless, trolox was unable to ameliorate the aforementioned effects completely and reduce the damage to control levels. Pizarro *et al*. [34] previously showed trolox’s capacity to inhibit ROS production mediated by 500 μM H_2_O_2_, which suppressed H_2_O_2_-induced cytotoxicity in neuroblastoma B65 cells. The higher antioxidant capacity of trolox compared with LMPS might have two reasons. First, the molecular weight of trolox (MW = 250.29 Da) is lower than that of LMAG (MW = 3573 Da), LMCH (MW = 3767 Da), and LMST (MW = 3643 Da). Thus, trolox exerts its action in solution faster than LMPS, because of a higher degree of freedom, thereby rendering trolox more efficient than LMPS in quenching ROS. Second, since trolox is a phenolic antioxidant (A–OH), it is able to terminate free radical species (R•), forming a stable phenoxyl radical (A–O•) by contributing hydrogen atoms from phenolic hydroxyl groups. Phenolic compounds act via hydrogen donation and subsequent radical stabilization by delocalizing the free electron over a conjugated double-bond system [35]. In contrast, the internal pyranose ring in LMPS contains no conjugated double bonds to stabilize the radical. Furthermore, LMCH, LMAG, and LMST contain many hydroxyl groups in their structure, but these would not act like phenolic compounds do, by donating hydrogen atoms to free radicals to terminate free radical species. This is most likely the reason why the cell viabilities were lower and the ROS production, and concomitant biomolecule damage was higher in LMCH-, LMAG-, and LMST-treated samples compared with trolox-treated ones.

**Scheme 1:**
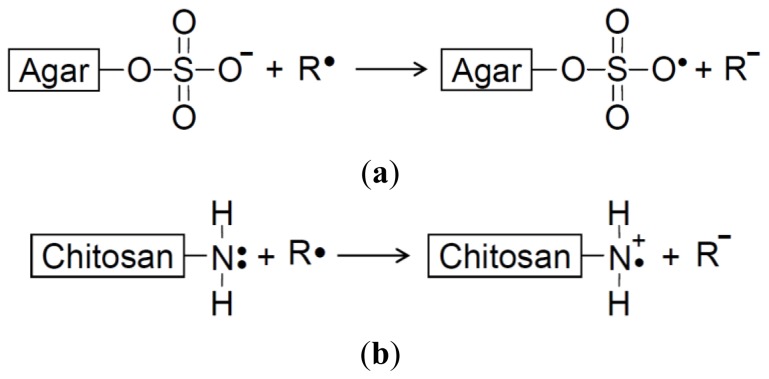
The probable mechanism of electron transfer between (**a**) low-molecular-weight agar and the free radical (R•); (**b**) low-molecular-weight chitosan and the free radical to form the stable LMPS radical (at pH = 7.2 in DMEM culture medium).

**Scheme 2:**
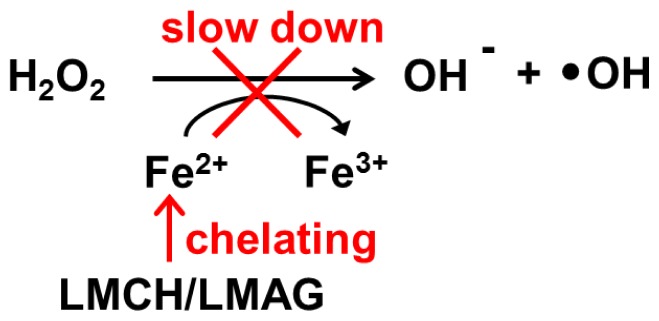
The metal ion chelating effect of LMCH and LMAG.

In summary, the cell viabilities were: trolox > LMCH > LMAG > LMST (Figure 2). The extent of ROS production, lipid peroxidation, and DNA damage in Hs68 cells were: LMCH < LMAG < LMST < trolox (Figures 3–5), in agreement with the cell viability. The results indicated that LMCH had a significantly higher capacity to protect HS68 cells from oxidative damage induced by H_2_O_2_ than LMAG and LMST. This may be due to the following reasons:

(1) Electron transfer effect: The antioxidative capacities of LMCH and LMAG might be due to scavenging of free radicals via electron transfer to form stable macromolecular radicals through the amino and sulfate groups, respectively (Scheme 1) [17]. In other words, the electron transfer from the sulfate group of LMAG to free radicals (R•) to form a stable LMAG radical (Scheme 1A) may function in the same way as the electron transfer from the amine group of LMCH to free radicals (R•) to form a stable LMCH radical (Scheme 1B). The electron cloud density of a functional group affects its electron-donating activity. High electron cloud densities increase the electron-donating activity, and this increases the free radical scavenging ability and the reducing power [24]. The size of the electron cloud density of a sulfate group is larger than that of the amine group, and this may explain the larger electron transfer effect by LMAG compared with LMCH.
(2) Metal ion-chelating effect: LMCH might chelate metal ions through the chelating ligands by amino groups and hydroxyl groups at position C-2 and C-3, respectively. Conversely, LMAG might chelate metal ions by electrostatic interaction between the negative charge of the sulfate groups and the metal ions (Scheme 2). The effect of LMCH’s chelating capacity is greater than the electrostatic interaction between the negative charge of LMAG’s sulfate groups and metal ions [17].
(3) Hydrophobic effect: The glucosamine and *N*-acetylglucosamine in LMCH is less hydrophobic than the 3,6-anhydro-galactose residue in LMAG, facilitating access to free radicals in LMCH [17], which results in a significantly higher free radical scavenging capacity of LMCH compared with LMAG.

To summarize the three aforementioned effects: the high free radical scavenging and metal ion sequestering properties of LMCH result in a high antioxidative capacity that is superior to those of LMAG. Consequently, LMCH displayed a high capacity to limit ROS production (Figure 3), inhibited cellular lipid peroxidation (Figure 4), and protected against oxidative DNA damage (Figure 5). Conversely, the hydroxyl groups in LMST have no charged groups or chelating ligands to chelate the metal ions and transfer electrons to free radicals, respectively. Furthermore, the internal pyranose ring in LMST has no conjugated double bonds to delocalize the free electron and stabilize the radical. Together, these reasons explain why LMST was incapable of limiting ROS production and protecting HS68 cells against oxidative damage.

## 4. Experimental Section

### 4.1. Polysaccharide Materials

Agar (Cat. No. 1802; Lot no. HB 0112640-A) was purchased from Laboratorios, S. A. (Madrid, Spain). Chitosan (MW = 500 kDa; DD = 90%) was obtained from Lytone Enterprise, Inc. (Taipei, Taiwan). Starch (Cat. No. 18727; Lot no. 53610) was purchased from Sigma-Aldrich Co. (St. Louis, MO, USA).

### 4.2. Preparation of LMPS

Agar and starch were hydrolyzed for 4 h in 0.1 N HCl (10 g/L) under stirring at 60 °C. After acidic hydrolysis, the reaction was terminated by neutralization with 0.1 M NaOH in an ice-water bath. The hydrolysates were obtained by centrifuging at 6000 × *g* for 15 min, and subsequently filtered to remove insoluble particles. Chitosan was suspended in 10% hydrogen peroxide (10 g/100 mL) at 60 °C under stirring for 4 h. The mixture was centrifuged at 6000 × *g* for 15 min, and filtered to remove insoluble particles. Agar, starch, and chitosan hydrolysis solutions were desalted by dialyzing against distilled water using 1000 Da MW cut-off dialysis membranes (Membrane Filtration Products, Inc., Seguin, TX, USA), and then filtered by 5000 Da MW ultrafilter membranes (Millipore Co., Billerica, MA, USA). The filtrate was concentrated by rotary evaporation and lyophilized to obtain samples of LMPS.

### 4.3. Molecular Weight Determination

The MWs of LMPS were determined by size-exclusion high-performance liquid-chromatography [36], using a column packed with TSK gel G4000 PW_XL_ and G5000 PW_XL_ (Tosoh Co., Ltd., Tokyo, Japan). The elution peak was detected with a Gilson (Middleton, WI, USA) M132 RI detector. The MWs of the samples were calculated from the pullulan standards (Shodex, Kawasaki, Japan) calibration curve with Chem-Lab software (Scientific Information Service Co., Taipei, Taiwan).

### 4.4. Degree of Deacetylation Measurements

Infrared spectrometry was used to determine the degree of deacetylation (DD) of the chitosans [37]. LMCH powder was mixed with KBr (1:100) and pressed into a pellet. The absorbances of amide 1 (1655 cm^−1^) and of the hydroxyl band (3450 cm^−1^) were measured using a Bio-Rad FTS-155 infrared spectrophotometer. The band of the hydroxyl group at 3450 cm^−1^ was used as an internal standard to correct for disc thickness and for differences in chitosan concentration during KBr disc preparation. The percentage of the amine group’s acetylation in the sample is given by (A_1655_/A_3450_) × 115. Where A_1655_ and A_3450_ are the absorbances at 1655 cm^−1^ and 3450 cm^−1^, respectively.

### 4.5. Sulfate Content Measurements

The sulfate content was determined via the rhodizonate method [38] with a sulfate standard. Briefly, 0.5 mL of an aqueous LMPS solution (100 μg/mL) was mixed with 0.5 mL HCl (1 N), and heated at 100 °C for 1 h. The mixture was dried by evaporation at 65 °C; then 0.5 mL distilled water was added to prepare a hydrolyzed LMPS solution. Subsequently, 0.5 mL of the hydrolyzed LMPS solution was mixed with 2 mL ethanol (95%), 1 mL BaCl_2_ solution (10 mL of 2 M acetic acid, 0.2 mL of 0.05 M BaCl_2_, and 0.8 mL of 0.02 M NaHCO_3_; diluted to 100 mL with 95% ethanol), and 1.5 mL sodium rhodizonate reagent (1 mg sodium rhodizonate and 10 mg ascorbate dissolved in 20 mL de-ionized water; diluted to 100 mL with 95% ethanol). The mixture was shaken and allowed to stand at room temperature in the dark. After 20 min, the absorbance was measured spectrophotometrically at 520 nm.

### 4.6. Cell Culture

Human foreskin fibroblast (Hs68) cells were obtained from the Bioresource Collection and Research Center (Hsinchu, Taiwan). Hs68 cells were grown in Dulbecco’s Modified Eagle Medium (DMEM), containing 10% (*v*/*v*) fetal bovine serum (FBS), 4 mM l-glutamine, 100 U/mL penicillin and 100 μg/mL streptomycin, and incubated at 37 °C in a humidified atmosphere (5% CO_2_). The 7th to 10th passages of Hs68 cells were used for experiments after unfreezing.

### 4.7. Cytotoxicity Determination

The cytotoxic effects of the various samples on cells were measured using the MTT (3-(4,5-dimethylthiazol-2-yl)-2,5-diphenyltetrazolium bromide) assay as described by Ngo *et al*. [25]. Hs68 cells were seeded in 96-well plates at an initial density of 5 × 10^3^ cells/well. After 24 h, the culture medium was replaced with fresh medium containing various concentrations of LMAG, LMCH, LMST, or trolox. Culture medium only was used as a control. After 24 h of incubation, 20 μL MTT (5 mg/mL) was added and incubated for 4 h. Finally, the culture medium with MTT was replaced with 200 μL DMSO to solubilize the formazan salt formed. After 10 min, the optical density (*OD*) of the formazan salt was measured at 570 nm with a Synergy Mx series microplate reader (BioTek, VT, USA). The viabilities of cells treated with various concentrations of LMPS and trolox samples were calculated according to the following equation:

(1) $$\text{Cell viability }(\%)=\frac{{\text{OD}}_{\text{sample}}}{{\text{OD}}_{\text{control}}}\times 100$$

where *OD*_control_ is the absorbance value of the control and *OD*_sample_ is the absorbance value of the samples.

### 4.8. Oxidative Stress Induction

Hs68 cells were cultured in 24-well plates at an initial density of 2 × 10^4^ cells/well and incubated for 24 h before treatment. Subsequently, the culture medium was replaced with fresh medium containing LMAG, LMCH, LMST, or trolox. After 2 h of incubation, H_2_O_2_ was added in a series of wells (final concentration 500 μM) to induce oxidative stress. Culture medium without H_2_O_2_ treatment was used as a control. After 24 h of oxidative stress treatment, the cells’ viability was determined via the MTT assay.

### 4.9. Intracellular ROS Determination

Intracellular ROS production was measured by detecting the fluorescence intensity of the oxidation-sensitive dye 2′,7′-dichlorofluorescin diacetate (DCFH-DA) [25]. Hs68 cells were cultured in fluorescence microtiter 96-well plates at an initial density of 10^4^ cells/well. Subsequently, the culture medium was replaced with fresh medium containing 20 μM DCFH-DA to label the Hs68 cells for 45 min. After removing the medium and washing the cells with PBS three times, the culture medium was replaced by fresh medium containing LMAG, LMCH, LMST, or trolox. Culture medium without H_2_O_2_ treatment was used as a control. After 2 h of incubation, H_2_O_2_ was added in a series of wells (final concentration 500 μM) to induce oxidative stress for 30 min. The intensity of the fluorescence signal emitted by 2′,7′-dichlorofluorescin (DCF) was measured at an excitation wavelength of 485 nm and emission wavelength of 528 nm with a BioTek Synergy Mx series microplate reader. The ROS production of the cells was calculated according to the following equation:

(2) $$\text{ROS production }(\%)=\frac{{\text{F}}_{\text{sample}}-{\text{F}}_{\text{control}}}{{\text{F}}_{\text{control}}}\times 100$$

where *F*_control_ is the fluorescence intensity of the control and *F*_sample_ is the fluorescence intensity of the samples.

### 4.10. Cellular Lipid Peroxidation Determination

Cellular lipid peroxidation in Hs68 cells was assayed according to the method described by Chiou *et al*. [39]. Hs68 cells were seeded in 10 cm tissue culture dishes at an initial density of 2 × 10^6^ cells and cultured for 24 h before treatment. Subsequently, the culture medium was replaced with fresh medium containing LMAG, LMCH, LMST, or trolox. After 2 h of incubation, H_2_O_2_ was added (final concentration 500 μM) to induce oxidative stress. Culture medium without H_2_O_2_ treatment was used as a control. After 24 h of oxidative stress treatment, the cells were washed three times with PBS, scraped from the dish and then dispersed in 2 mL PBS containing 0.5 mM BHT (butylatedhydroxytoluene). The cell suspensions were transferred to centrifugal tubes and 2 mL 15% TCA (trichloroacetic acid) and 2 mL 0.7% TBA (thiobarbituric acid) was added. After being mixed thoroughly, the solution was then heated for 15 min in a boiling water bath. After cooling, the insoluble precipitates were removed by centrifugation at 10,000 × *g* for 10 min. The absorbance was measured spectrophotometrically at 560 nm. The lipid peroxidation level (%) was calculated as follows:

(3) $$\text{Lipid peroxidation level }(\%)=\frac{{\text{A}}_{\text{sample}}-{\text{A}}_{\text{control}}}{{\text{A}}_{\text{control}}}\times 100$$

where *A*_control_ is the absorbance of the control, and *A*_sample_ is the absorbance of samples.

### 4.11. DNA Damage Determination

DNA damage of the sample cells was measured using the Comet assay (Single Cell Gel Electrophoresis), as described by Chen and Wong [40]. Hs68 cells were cultured in 6-well plates at an initial density of 1 × 10^5^ cells/well. After 24 h, the culture medium was replaced with fresh medium containing LMAG, LMCH, LMST, or trolox. Culture medium without H_2_O_2_ treatment was used as a control. After the medium was removed and the cells were washed with PBS three times, the culture medium was replaced with fresh medium containing LMAG, LMCH, LMST, or trolox. After 2 h of incubation, H_2_O_2_ was added in a series of wells (final concentration 500 μM) to induce oxidative stress for 30 min. After oxidative stress treatment, the cells were washed three times with PBS, scraped from 6-well plates and then dispersed in 1 mL PBS. A CometAssay^™^ reagent kit for single-cell gel electrophoresis (Trevigen, Inc., Gaithersburg, MD, USA) was used to detect DNA damage according to the manufacturer’s protocol. Briefly, the cell suspension was combined with molten LMAG at a ratio of 1:10 (*v*/*v*) at 37 °C; 50 μL were immediately pipetted onto the CometSlides^™^, which were subsequently kept horizontally at 4 °C in the dark for 10 min until clear rings appeared at the edge of the slide area. The slides were then immersed in pre-cooled lysis solution (containing 10% DMSO) at 4 °C for 30 min. This was followed by immersion in a freshly prepared alkaline solution (300 mM NaOH, 1 mM EDTA, pH > 13) for 30 min in the dark. Slides were removed from the alkaline solution, excess buffer was gently drained from the slide, and the slides were washed by immersing them twice in 1× TBE buffer (Tris-borate-EDTA buffer) for 5 min. Slides were placed in an electrophoresis tank (CometAssay^™^ tank, Trevigen, Inc., Gaithersburg, MD, USA) with 1× TBE buffer and the power supply was set to 21 V for 10 min. After electrophoresis, slides were dipped into de-ionized water several times, then immersed in 70% ethanol for 5 min, and dried in the air. Finally, DNA was stained with diluted SYBR^®^ Green I (Trevigen, Inc., Gaithersburg, MD, USA) in the refrigerator for 5 min. The slides were then tapped gently to remove excess SYBR^®^ solution and were allowed to dry completely at room temperature in the dark. The slides were viewed immediately on an Eclipse 80i fluorescence microscope (Nikon Corporation, Tokyo, Japan) and images were captured with a digital camera (Coolpix E5400, Nikon, Tokyo, Japan). Fifty cells per slide were selected randomly, and a CometScore V1.5 image analysis system (TriTek Corp., Sumerduck, VA, USA, 2006) was used to measure the level of DNA damage, which was expressed as a percentage of tail DNA, which represents the amount of DNA present in the tail due to strand breaks.

### 4.12. Statistical Analysis

All results are expressed as mean ± SD of triplicate experiments (*n* = 3). Data were analyzed via a one-way analysis of variance (ANOVA). When the ANOVA identified differences among the groups, multiple comparisons among means were made using Duncan’s new multiple range test. Statistical significance was determined by setting the aggregate type I error at 5% (*p* < 0.05), for each set of comparisons, using Statistical Analysis System software (SAS 8.0, SAS Institute Inc., Cary, NC, USA, 1999).

## 5. Conclusions

Our results showed that LMCH and LMAG afforded protection against H_2_O_2_-induced oxidative stress by enhancing cell viability and inhibiting intracellular ROS production, lipid peroxidation, and oxidative DNA damage in skin fibroblast. The differences in protection against oxidative stress in skin fibroblasts may be due to the combined effects of electron transferability, delocalization of the free electron, metal ion chelating capacities, and the accessibility of LMPS to ROS, which in turn depends on the different hydrophobicities of the constituent sugars. The antioxidative mechanisms in cells are more complex than in clean chemical experimental environments. Many endogenous and environmental factors, such as ultraviolet light, metal ions, chemical reactions, intracellular antioxidant enzymes and cytokines, influence the generation of ROS and induction of oxidative stress in cells and tissues. The results obtained here *in cellulo* are more biologically relevant than those previously reported in simple and clean *in vitro* experiments. The current results may contribute to develop novel therapeutic interventions to counteract skin aging related as a consequence of ROS generation by UV radiation generating ROS. However, this will require further *in vivo* investigations in physiologically relevant models, such as a “skin equivalent model” or lab animals. Nonetheless, future applications of LMAG and LMCH may lie in cosmetics and dermatological therapeutics to scavenge ROS and retard skin aging.
